# Distinct profiles of osteoclast and dendritic cell-mediated expansion and functional activation of NK and T cells

**DOI:** 10.1007/s00262-025-03956-x

**Published:** 2025-03-01

**Authors:** Kawaljit Kaur, Anahid Jewett

**Affiliations:** 1 Division of Oral Biology and Medicine, The Jane and Jerry Weintraub Center for Reconstructive Biotechnology, University of California School of Dentistry, 10833 Le Conte Ave., Los Angeles, CA 90095 USA; 2 The Jonsson Comprehensive Cancer Center, UCLA School of Dentistry and Medicine, 10833 Le Conte Ave., Los Angeles, CA 90095 USA

**Keywords:** NK cells, T cells, CD4 + T cells, CD8 + T cells, Osteoclasts, Dendritic cells, IFN-γ

## Abstract

Osteoclasts (OCs) and dendritic cells (DCs) induce expansion and functional activation of NK and T cells. When comparing OCs with DC-induced activation in NK cells, OCs induced significantly higher cell expansion and functional activation of NK cells as compared to DCs, either from healthy individuals or those obtained from cancer patients. However, no differences could be seen in the levels of cell expansion and functional activation in T cells activated by OCs or DCs, either from healthy individuals or those from cancer patients. OCs selectively expanded and activated CD8 + T cells, whereas DCs expanded and activated CD4 + T cells. In addition, both allogeneic and autologous OCs induced similar levels of cell expansion and functional activation of NK and T cells. Together, these findings highlighted the essential roles of OCs in expanding and activating the cytotoxic effectors of NK, and CD8 + T cells, and demonstrated several differences when compared to the effect of DCs.

**Supplementary Information:**

The online version contains supplementary material available at 10.1007/s00262-025-03956-x.

## Introduction

Osteoclasts (OCs) are primarily known for their role in bone breakdown or resorption. Natural killer (NK) and T cells are among the large populations of lymphocytes in peripheral blood mononuclear cells (PBMCs). NK cells originate from bone marrow and are known to regulate the functions of other immune cells by producing key cytokines and chemokines [1, 2]. NK cell-mediated cytotoxicity results in the lysis of transformed or infected cells, and also NK cells limit tumor growth/metastasis and viral infections [3]. Decreased numbers and cytotoxic activity of NK cells result in poor prognosis in cancer patients [4–9]. T cells mediate adaptive cellular immunity [10, 11]. CD8 + T cells play a significant role in immune surveillance and defense against infections and cancer [12, 13]. Increased number of tumor-infiltrating CD8 + T cells are associated with positive responses to standard chemotherapeutic regimens and patient survival [14–17]. CD4 + T cells mediate effector function via secreted cytokines [17]. CD4 + and CD8 + T cells mediated adaptive cellular immunity closely collaborate with the innate immune system [10, 11]. It was found that NK cells can activate and induce the proliferation of T cells through direct cell–cell contact [18–20]. The immunoregulatory function of NK cells plays a role in killing chronically activated leukocytes [21–24] and eliminating activated autologous CD4 + T cells [24–26].

We have previously explored the immunomodulatory function of OCs and demonstrated the role of OCs in the modulation of NK cells, and T cells [27–32]. OCs were found to play a crucial role in the expansion and functional activation of NK cells and CD8 + T cells [29, 30, 33, 34]. Since OCs preferentially expand CD8 + T cells, OC-induced expanded T cells expressed decreased ratios of CD4 + to CD8 + T cells [30]. We previously have shown differences between dendritic cells (DCs)-induced expanded NK and T cells [30]. OCs induced higher expansion and functional activation of NK cells compared to DCs, preferentially expanded CD8 + T cells, whereas, DCs preferentially expanded CD4 + T cells [30]. The mechanisms governing the differential expansion of NK and T cells by OCs and DCs are still under investigation. These findings suggest that OCs are the best feeder cells to prepare NK and CD8 + T cell-based immunotherapies. To date, NK and CD8 + T cell-based immunotherapies are among the leading standards in cancer therapeutics [35, 36].

This study explores the differences between OCs and DCs-induced expansion and functional activation of NK and T cells. We compared the NK and T cell expansion and functional activation induced by healthy individual and cancer patient-derived OCs and DCs. In addition, we observed that similar levels of expansion and functional activation were induced in both NK and T cells using allogeneic or autologous healthy individual-derived OCs. Finally, we demonstrated that OCs expand and activate CD8 + T cells higher when compared to CD4 + T cells, whereas DC expands and activates CD4 + T cells higher when compared to CD8 + T cells.

## Materials and methods

### Cell lines, reagents, and antibodies

RPMI 1640 supplemented with 10% fetal bovine serum (FBS) (Gemini Bio-Product, CA, USA) was used to culture human NK cells and T cells. Alpha-MEM (Life Technologies, CA, USA) supplemented with 10% FBS was used for osteoclast (OCs) and dendritic cell (DCs) cultures. M-CSF, anti-CD16 mAb, and flow cytometric antibodies were purchased from Biolegend, CA, USA. RANKL, GM-CSF, and IL-4 were purchased from PeproTech, NJ, USA, and recombinant human IL-2 was obtained from Hoffman La Roche (NJ, USA). Human anti-CD3/CD28 was purchased from Stem Cell Technologies, Vancouver, Canada. Probiotic bacteria, AJ2 is a combination of eight different strains of gram-positive probiotic bacteria selected for their superior ability to induce optimal secretion of both pro-inflammatory and anti-inflammatory cytokines in NK cells [37]. RPMI 1640 supplemented with 10% FBS was used to re-suspend AJ2. Oral squamous carcinoma stem cells (OSCSCs) were isolated from patients with tongue tumors at UCLA [38–41]. OSCSCs were cultured in RPMI 1640 (Life Technologies, CA, USA) supplemented with 10% fetal bovine serum (FBS) (Gemini Bio-Product, CA, USA). Human ELISA kits for IFN-γ were purchased from Biolegend (San Diego, CA).

### Purification of human NK cells, T cells, and monocytes

Written informed consents were obtained from healthy donors and cancer patients as approved by the UCLA Institutional Review Board (IRB), all procedure were approved by UCLA-IRB and all methods were carried in accordance with UCLA-IRB guidelines and regulations. Peripheral blood mononuclear cells (PBMCs) were isolated from peripheral blood as described before [42]. Briefly, PBMCs were obtained after Ficoll-hypaque centrifugation and were used to isolate NK cells, T cells, CD4 + T cells, CD8 + T cells, and monocytes using the EasySep® Human NK cell, EasySep® Human T cell, EasySep® Human CD4 T, and EasySep® Human CD8 T cell, EasySep® Human monocytes enrichments kits, respectively, purchased from Stem Cell Technologies (Vancouver, BC, Canada). Isolated NK cells, T cells, CD4 + T cells, CD8 + T cells, and monocytes were stained with anti-CD16, anti-CD3, anti-CD4, anti-CD8, anti-CD14 antibodies, respectively, to measure the cell purity using flow cytometric analysis.

### Generation of human OCs and DCs

To generate OCs, monocytes were cultured in alpha-MEM media supplemented with M-CSF (25 ng/mL) and RANKL (25 ng/mL) for 21 days, media was replenished every three days. OCs were tested using TRAP staining to confirm multinucleated cells as described previously [27]. Monocytes were cultured in alpha-MEM media supplemented with GM-CSF (150 ng/mL) and IL-4 (50 ng/mL) for 7 days to generate DCs.

### Sonication of probiotic bacteria (AJ2)

AJ2 bacteria were weighed and re-suspended in RPMI 1640 medium containing 10% FBS at a concentration of 10 mg/ml. The bacteria were thoroughly vortexed, then sonicated on ice for 15 s at 6 to 8 amplitudes, sonicated samples were then incubated for 30 s on the ice, the cycle was repeated for five rounds. After every five rounds of sonication, we checked each sample under the microscope until at least 80% of bacterial walls were lysed. It was determined that approximately 20 rounds of sonication/incubation on ice were necessary to achieve complete sonication. Finally, the sonicated AJ2 (sAJ2) was aliquoted and stored at −80˚C until use.

### Expansion of NK cells and T cells

Human purified NK cells were activated with rh-IL-2 (1000 U/ml) and anti-CD16 mAbs (3 µg/ml) for 18–20 h before they were co-cultured with feeder cells (OCs or DCs) and sAJ2 (OCs:NK:sAJ2 or DCs:NK:sAJ2; 1:2:4) in RPMI 1640 medium containing 10% FBS. The medium was refreshed every three days with RPMI containing rh-IL-2 (1500 U/ml). Purified human T cells were activated with rh-IL-2 (100 U/ml) and anti-CD3 (1 µg/ml)/anti-CD28 (3 µg/ml) for 18–20 h before they were co-cultured with OCs or DCs and sAJ2 (OCs:T:sAJ2 or DCs:T:sAJ2; 1:2:4) in RPMI 1640 medium containing 10% FBS. The culture media was refreshed with rh-IL-2 (150 U/ml) every three days.

### Enzyme-linked immunosorbent assays (ELISAs)

Single ELISAs and multiplex assays were performed as previously described [42]. To analyze and obtain the cytokine and chemokine concentration, a standard curve was generated by either two- or three-fold dilution of recombinant cytokines provided by the manufacturer.

### ^51^Cr release cytotoxicity assay

The ^51^Cr release assay was performed as described previously [43]. Briefly, different numbers of effector cells were incubated with ^51^Cr–labeled target cells. After a 4-h incubation period, the supernatants were harvested from each sample and the released radioactivity was counted using the gamma counter. The percentage-specific cytotoxicity was calculated as follows:

$$\% {\text{ Cytotoxicity }} = \frac{{{\text{Experimental}}\;{\text{cpm }} - {\text{spontaneous}}\;{\text{cpm}}}}{{{\text{Total}}\;{\text{cpm }}{-}{\text{ spontaneous}}\;{\text{cpm}}}}$$

LU 30/10^6^ is calculated by using the inverse of the number of effector cells needed to lyse 30% of tumor target cells X100.

### Statistical analyses

All statistical analyses were performed using the GraphPad Prism-8 software. An unpaired or paired, two-tailed student’s t-test was performed for the statistical analysis of experiments with two groups. One-way ANOVA with a Bonferroni post-test was used to compare different groups for experiments with more than two groups. (n) denotes the number of human donors or mice for each experimental condition. Duplicate or triplicate samples were used in the in vitro studies for assessment. The following symbols represent the levels of statistical significance within each analysis: ***(*p*-value < 0.001), **(*p*-value 0.001–0.01), *(*p* value 0.01–0.05).

## Results

### Increased levels of cell expansion, cytotoxicity, and IFN-γ secretion in OC-activated NK cells in comparison to DC-activated NK cells

**Fig. 1:**
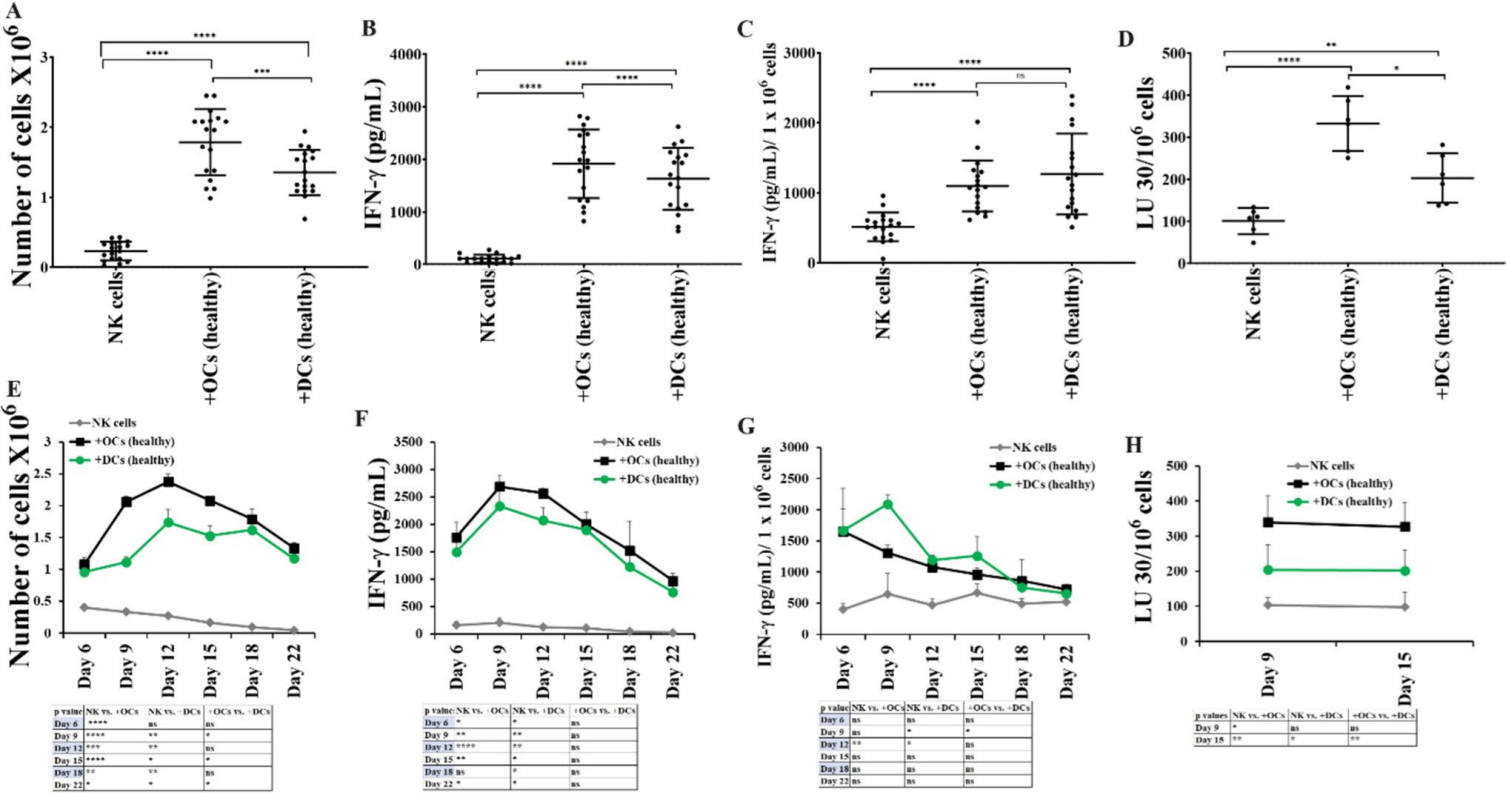
OCs induced higher cell expansion and functional activation in NK cells compared to DCs**.** OCs and DCs were generated as described in Materials and Methods. NK cells from healthy individuals (1 × 10^6^ cells/ml) were treated with a combination of IL-2 (1000 U/ml) and anti-CD16mAb (3 µg/ml) for 18 h before they were co-cultured with autologous OCs or DCs in the presence of sAJ2 at 1:2:4 ratios (DCs or OCs:NK:sAJ2). The expanding cells were counted on days 6, 9, 12, 15, 18, and 22 using microscopy (n = 18) **(A, E).** NK cells were co-cultured with OCs or DCs as described in Fig. 1A, the supernatants were harvested on days 6, 9, 12, 15, 18, and 22 of the co-cultures, and the amounts of IFN-γ secretion were determined using single ELISA (n = 18) **(B, F)**. The amounts of IFN-γ secretion shown in Fig. 1B and F were assessed based on 1 × 10^6^ cells (n = 18) **(C, G)**. NK cells were co-cultured with OCs or DCs as described in Fig. 1A and cytotoxicity of day 9 and 15 expanded cells was determined using a standard 4-h ^51^Cr release assay against OSCSCs. Effector-to-target (E:T) ratios were 2.5:1, 1.25:1. 0.65:1, and 0:1, and duplicate samples were used for each ratio. The Lytic units (LU) 30/10.^6^ cells were determined using the inverse number of NK cells required to lyse 30% of OSCSCs × 100 (n = 6) **(D, H).** ****(*p* value < 0.0001), ***(*p* value < 0.001), **(*p* value 0.001–0.01), *(*p* value 0.01–0.05)

To assess whether OCs and DCs induced different levels of cell expansion and functional activation in NK cells, we cultured NK cells from healthy individuals either alone or with OCs or DCs. When we counted the NK cells, a significantly increased level of cell expansion was observed in NK cells cultured with OCs compared to those cultured alone or with DCs (Fig. 1A, E). Cytokine secretion assay revealed significantly higher levels of IFN-γ in media harvested from NK cells and OCs co-culture compared to media from NK cells alone or NK cells and DCs cultures (Figs. 1B, C, F, G and S1). Also, NK cells cultured with OCs displayed significantly higher levels of NK cell-mediated cytotoxicity against oral squamous cancer stem-like cells (OSCSCs) compared to NK cells cultured alone or with DCs (Figs. 1D, 4H). Reduced surface expression of NK cell receptor ligands was seen on the surface of DCs compared to OCs (Fig. S2), this could be one of the mechanisms for reduced DCs induced activation in NK cells.

### Cancer patient-derived OCs and DCs exhibited decreased potential to induce cell expansion and functional activation in NK cells

**Fig. 2:**
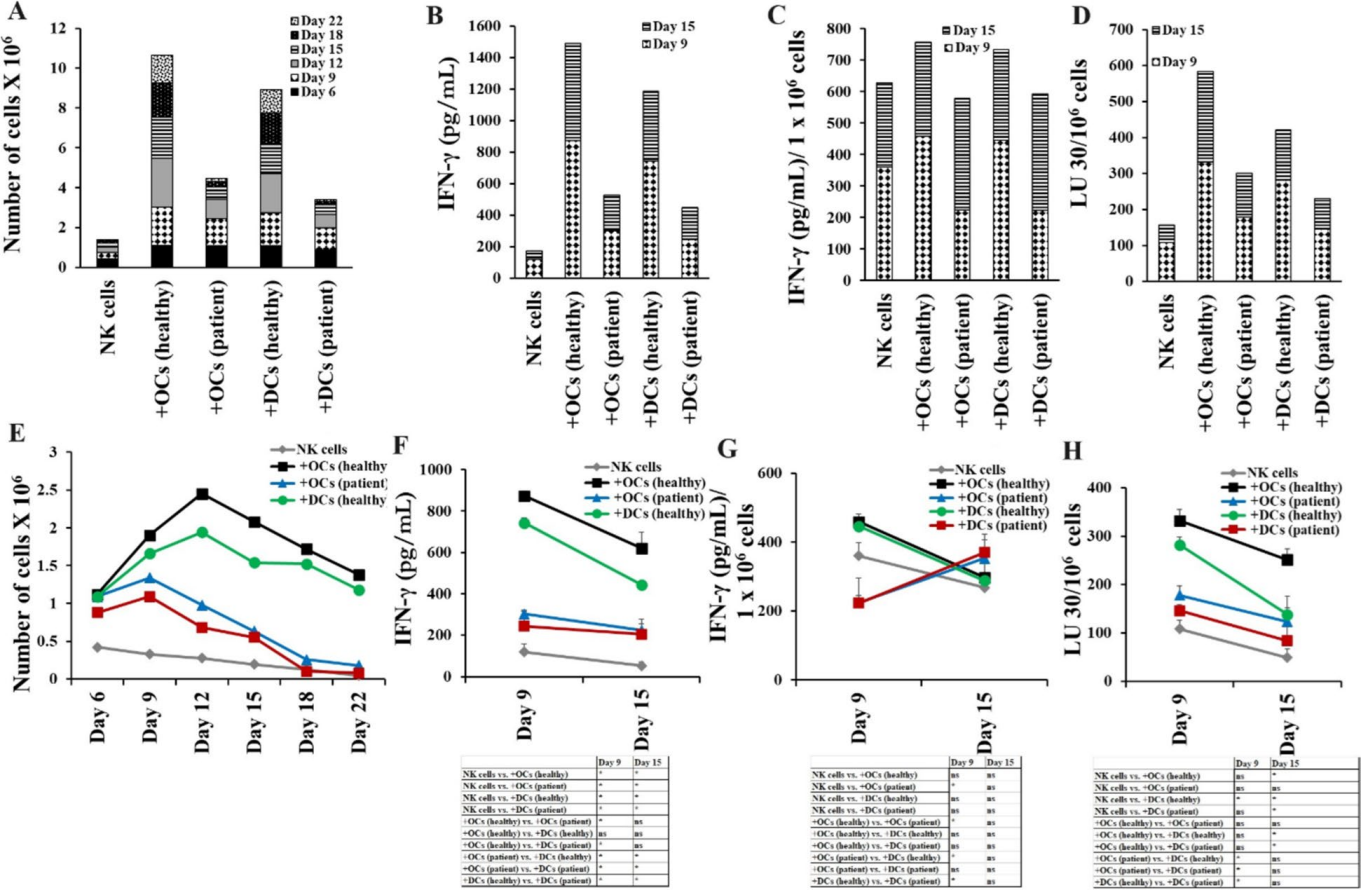
Both OCs and DCs from cancer patients induced lower cell expansion and functional activation in NK cells compared to healthy individual OCs and DCs, respectively. OCs and DCs were generated as described in Materials and Methods. NK cells from healthy individuals (1 × 10^6^ cells/ml) were treated with a combination of IL-2 (1000 U/ml) and anti-CD16mAb (3 µg/ml) for 18 h before they were co-cultured with allogeneic healthy individuals and cancer patients’ OCs or DCs in the presence of sAJ2 at 1:2:4 ratios (DCs or OCs:NK:sAJ2). The expanding cells were counted on days 6, 9, 12, 15, 18, and 22 using microscopy **(A, E).** NK cells were co-cultured with OCs or DCs as described in Fig. 2A, the supernatants were harvested on days 9 and 25 of the co-cultures, and the amounts of IFN-γ secretion were determined using single ELISA **(B, F)**. The amounts of IFN-γ secretion shown in Fig. 2B and F were assessed based on 1 × 10^6^ cells **(C, G)**. NK cells were co-cultured with OCs or DCs as described in Fig. 2A and cytotoxicity of day 9 and 15 expanded cells was determined using a standard 4-h ^51^Cr release assay against OSCSCs. Effector-to-target (E:T) ratios were 2.5:1, 1.25:1. 0.65:1, and 0:1, and duplicate samples were used for each ratio. The Lytic units (LU) 30/10.^6^ cells were determined using the inverse number of NK cells required to lyse 30% of OSCSCs × 100 **(D, H).** *(*p* value 0.01–0.05)

We cultured the healthy individuals’ NK cells with healthy individuals’ or pancreatic cancer patients’ derived OCs and DCs (allogeneic). The stage IV pancreatic cancer patient was a 53-year-old female with metastatic pancreatic adenocarcinoma (PDAC). She received several different chemotherapeutic drugs in various combinations over two years which included 12 cycles of Folfirinox, and anti-IL6R antibody therapy twice before her samples were used in this study. Healthy individuals with no medical history of cancer were age and gender-matched as cancer patients. Decreased levels of cell expansion (Fig. 2A, E), IFN-γ secretion (Figs. 2B–C, F–G, and S3), and NK cell-mediated cytotoxicity (Fig. 2D, H) were seen in NK cells cultured with patient-derived OCs and DCs in comparison to healthy donor-derived OCs and DCs, respectively. Reduced NK cell receptors on primary and OC-expanded NK cells (Figs. S4–S5), and their ligands on OCs’ surface were observed in cancer patient OCs (Fig. S6). These results indicated functional defects in cancer patient-derived NK cells, OCs, and DCs to induce optimal levels of cell expansion and activation in NK cells.

### Similar levels of cell expansion and functional activation induced by allogeneic or autologous OCs

**Fig. 3:**
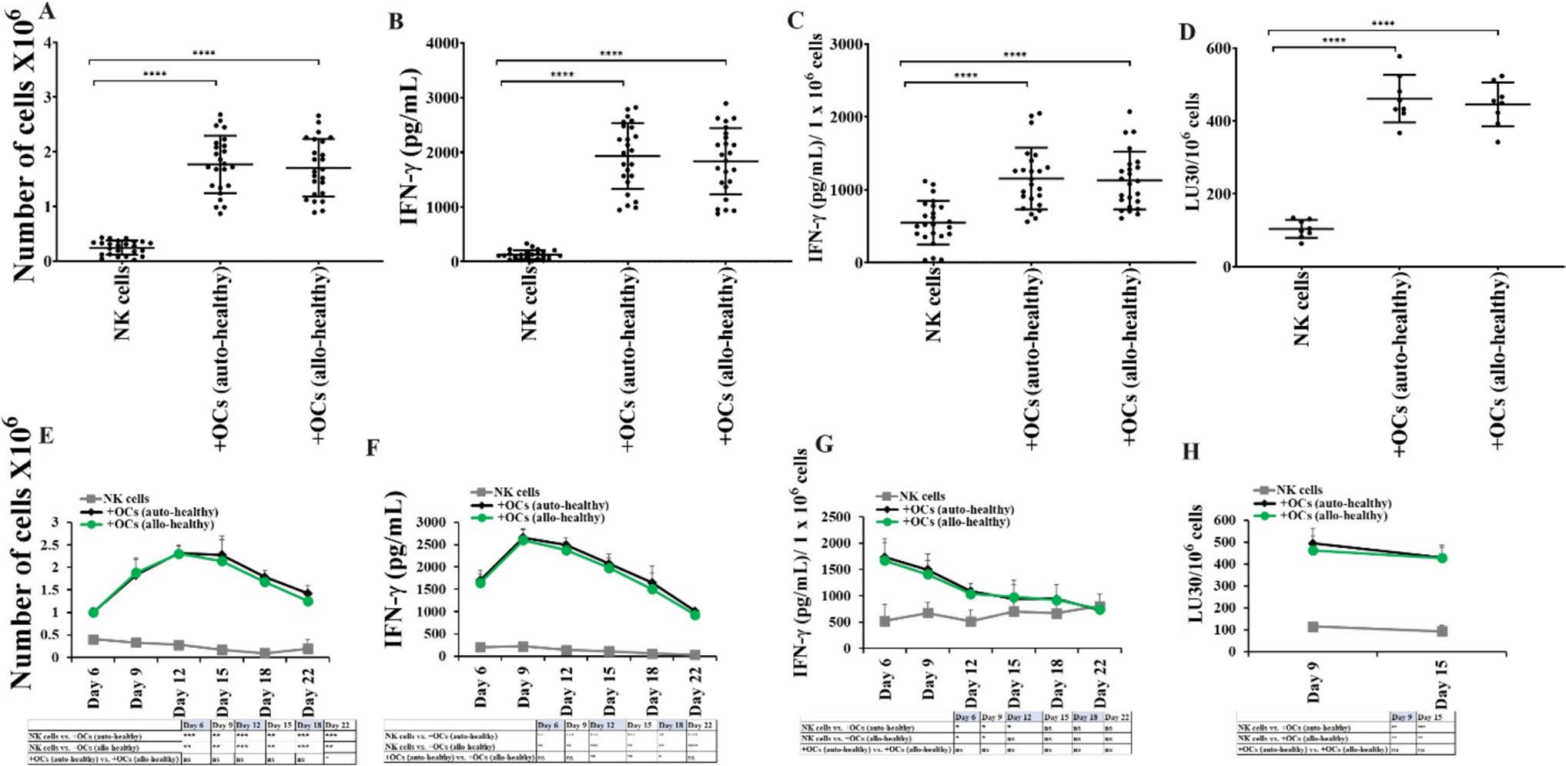
Autologous or allogeneic OCs from healthy individuals induced similar levels of cell expansion and functional activation in NK cells. OCs and DCs were generated as described in Materials and Methods. NK cells from healthy individuals (1 × 10^6^ cells/ml) were treated with a combination of IL-2 (1000 U/ml) and anti-CD16mAb (3 µg/ml) for 18 h before they were co-cultured with either autologous or allogeneic healthy individual derived OCs in the presence of sAJ2 at 1:2:4 ratios (OCs:NK:sAJ2). The expanding cells were counted on days 6, 9, 12, 15, 18, and 22 using microscopy (n = 24) **(A, E).** NK cells were co-cultured with OCs as described in Fig. 3A, the supernatants were harvested on days 6, 9, 12, 15, 18, and 22 of the co-cultures, and the amounts of IFN-γ secretion were determined using single ELISA (n = 24) **(B, F)**. The amounts of IFN-γ secretion shown in Fig. 3B and F were assessed based on 1 × 10^6^ cells (n = 24) **(C, G)**. NK cells were co-cultured with OCs as described in Fig. 3A and cytotoxicity of day 9 and 15 expanded cells was determined using a standard 4-h ^51^Cr release assay against OSCSCs. Effector-to-target (E:T) ratios were 2.5:1, 1.25:1. 0.65:1, and 0:1, and duplicate samples were used for each ratio. The Lytic units (LU) 30/10.^6^ cells were determined using the inverse number of NK cells required to lyse 30% of OSCSCs × 100 (n = 8) **(D, H).** ****(*p* value < 0.0001), ***(*p* value < 0.001), **(*p* value 0.001–0.01), *(*p* value 0.01–0.05)

To determine whether OC-induced activation in NK cells is donor-dependent, we cultured the NK cells from healthy individuals with either autologous or allogeneic healthy individual-derived OCs. Similar levels of cell expansion (Fig. 3A, E), IFN-γ secretion (Fig. 3B, C, F, G), and NK cell-mediated cytotoxicity (Fig. 3D, H) were observed in NK cells cultured with either autologous or allogeneic OCs.

### Not much difference was seen in the levels of cell expansion or IFN-γ secretion in T cells activated with OCs or DCs

**Fig. 4:**
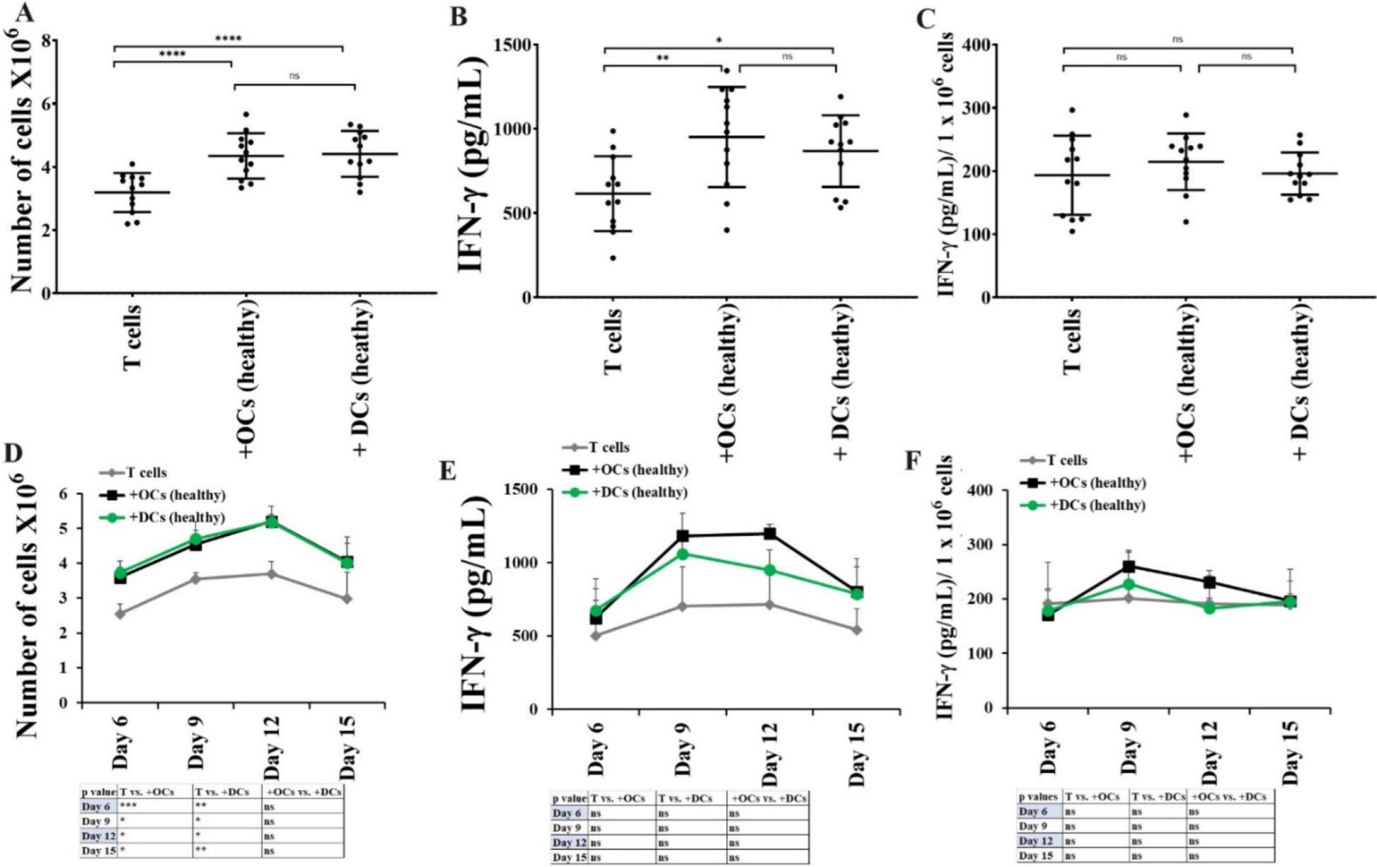
Both OCs and DCs from healthy individuals induced almost similar levels of cell expansion and function activation in T cells. T cells (1 × 10^6^ cells/ml) from healthy individuals were treated with a combination of IL-2 (100 U/ml) and anti-CD3 (1 µg/ml)/CD28mAb (3 µg/ml) for 18 h before they were co-cultured with healthy individuals’ OCs or DC and sAJ2 at a ratio of 1:2:4 (OCs or DCs:T:sAJ2). On days 6, 9, 12, and 15, the T cells were counted using microscopy (n = 12) **(A, D)**. T cells were treated and cultured as described in Fig. 4A and D. The supernatants were harvested on days 6, 9, 12, and 15, and the levels of IFN-γ secretion were determined using a single ELISA (n = 12) **(B, E)**. Amounts of IFN-γ secretion shown in Fig. 4B and E were assessed based on 1 × 10.^6^ cells (n = 12) **(C, F)**. ****(*p* value < 0.0001), ***(*p* value < 0.001), **(*p* value 0.001–0.01), *(*p* value 0.01–0.05)

Next, we analyzed OCs vs. DCs-induced cell expansion and functional activation in T cells. T cells from healthy individuals were cultured with OCs or DCs, and no detectable differences were seen in the levels of cell expansion (Fig. 4A, D), and IFN-γ secretion (Fig. 4B, C, E, F) in T cells in the presence of OCs vs. DCs. These results suggested that both OCs and DCs exhibit similar levels of potential to induce activation in T cells. OC-expanded T cells showed increased percentages of CD8 + T cells, whereas DC-expanded T cells showed increased percentages of CD4 + T cells (Fig. S7).

### Function inhibition in T cells from cancer patients was not significantly revered in the presence of either OCs or DCs

**Fig. 5:**
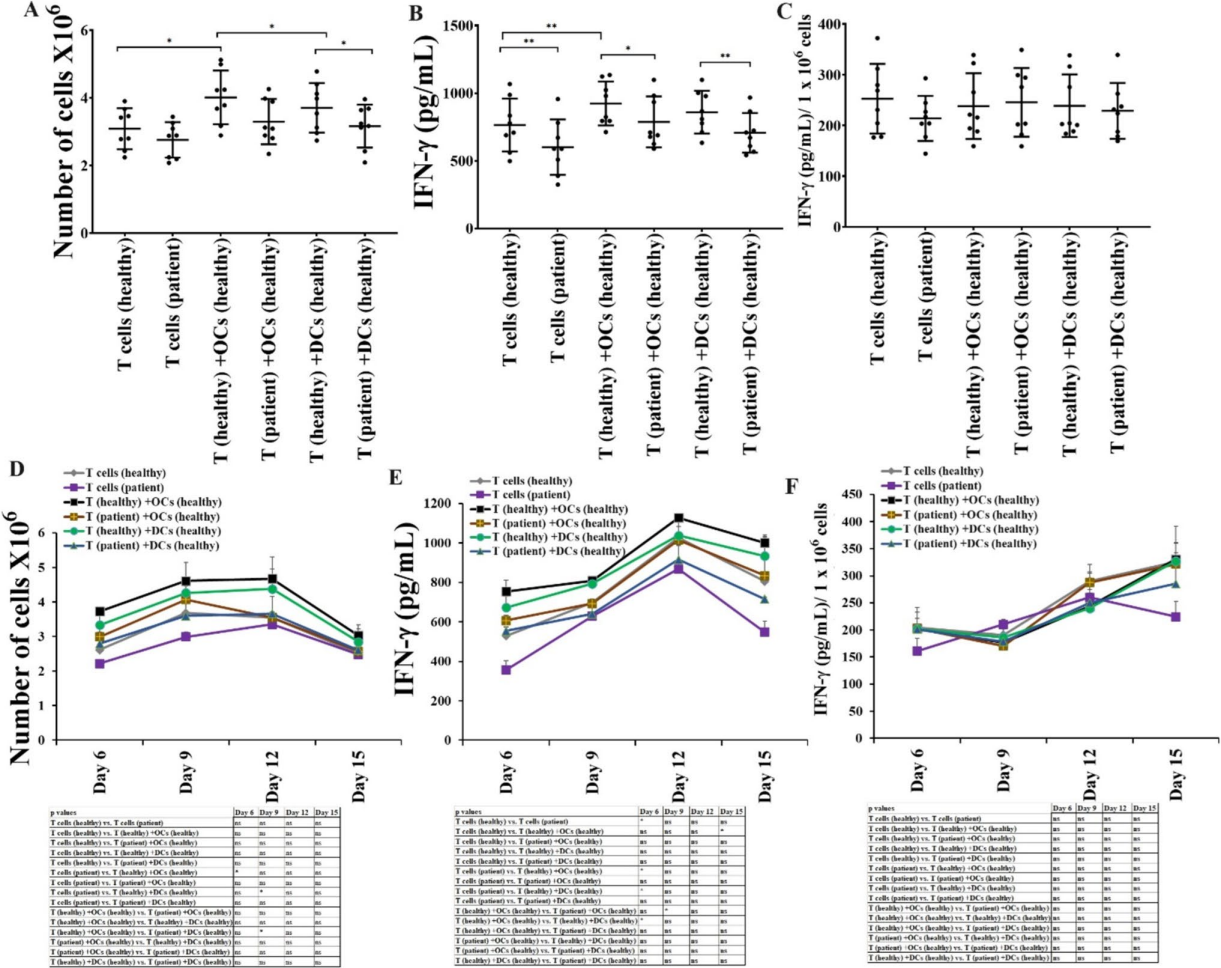
Both OCs and DCs from healthy individuals induced lower levels of cell expansion and function activation in cancer patient-derived T cells in comparison to T cells from healthy individuals. T cells (1 × 10^6^ cells/ml) from healthy individuals and cancer patients were treated with a combination of IL-2 (100 U/ml) and anti-CD3 (1 µg/ml)/CD28mAb (3 µg/ml) for 18 h before they were co-cultured with healthy individuals’ OCs or DC and sAJ2 at a ratio of 1:2:4 (OCs or DCs:T:sAJ2). On days 6, 9, 12, and 15, the T cells were counted using microscopy (n = 8) **(A, D)**. T cells were treated and cultured as described in Fig. 5A and D. The supernatants were harvested on days 6, 9, 12, and 15, and the levels of IFN-γ secretion were determined using a single ELISA (n = 8) **(B, E)**. Amounts of IFN-γ secretion shown in Fig. 5B and E were assessed based on 1 × 10.^6^ cells (n = 8) **(C, F)**. **(*p* value 0.001–0.01), *(*p* value 0.01–0.05)

**Fig. 6:**
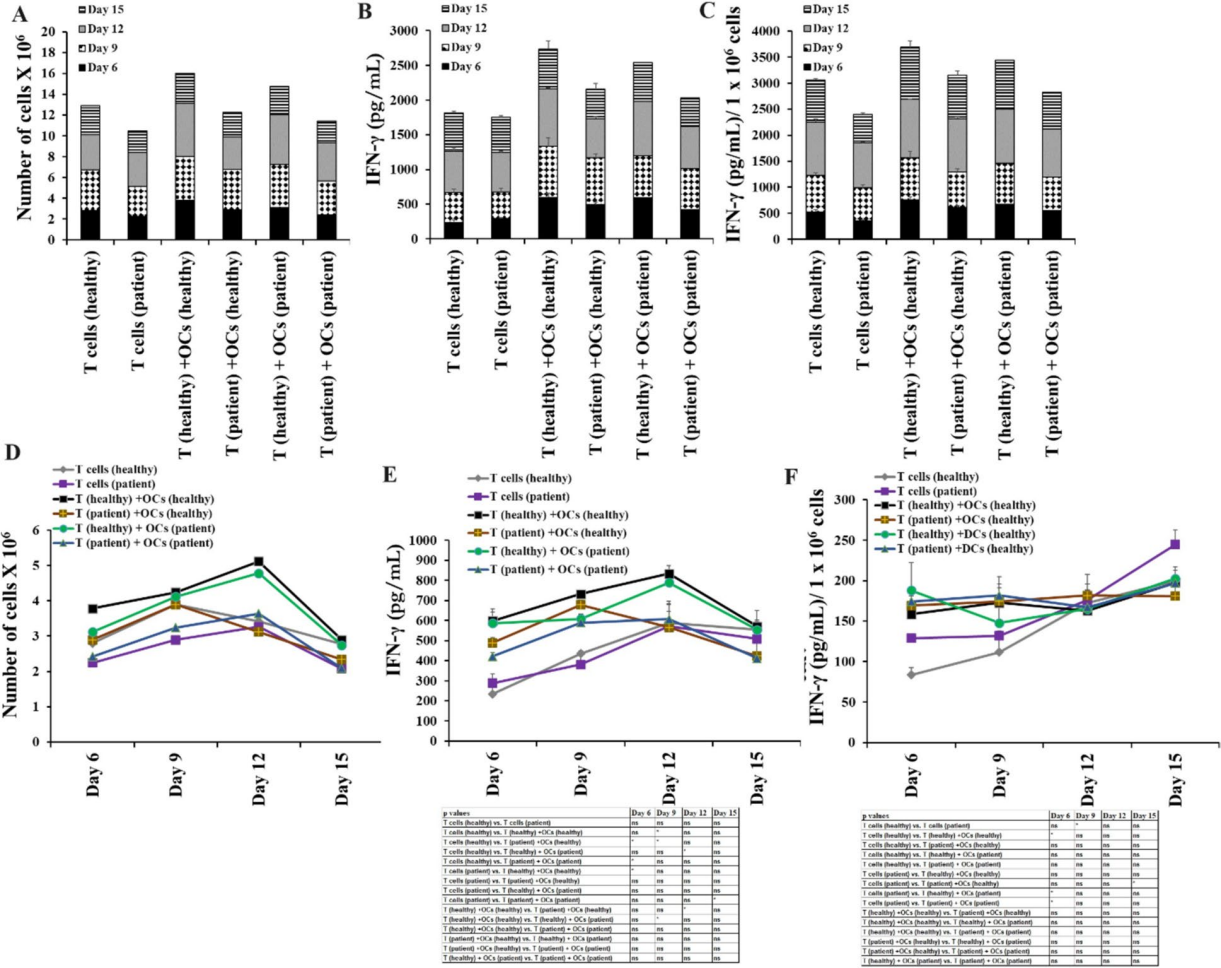
OCs from cancer patients induced lower levels of cell expansion and function activation in both cancer patient and healthy individual-derived T cells in comparison to OCs from healthy individuals. T cells (1 × 10^6^ cells/ml) from healthy individuals and cancer patients were treated with a combination of IL-2 (100 U/ml) and anti-CD3 (1 µg/ml)/CD28mAb (3 µg/ml) for 18 h before they were co-cultured with healthy individuals’ or cancer patients’ OCs and sAJ2 at a ratio of 1:2:4 (OCs:T:sAJ2). On days 6, 9, 12, and 15, the T cells were counted using microscopy **(A, D)**. T cells were treated and cultured as described in Fig. 6A and D. The supernatants were harvested on days 6, 9, 12, and 15, and the levels of IFN-γ secretion were determined using a single ELISA **(B, E)**. Amounts of IFN-γ secretion shown in Fig. 6B and E were assessed based on 1 × 10.^6^ cells **(C, F)**. **(*p* value 0.001–0.01), *(*p* value 0.01–0.05)

**Fig. 7:**
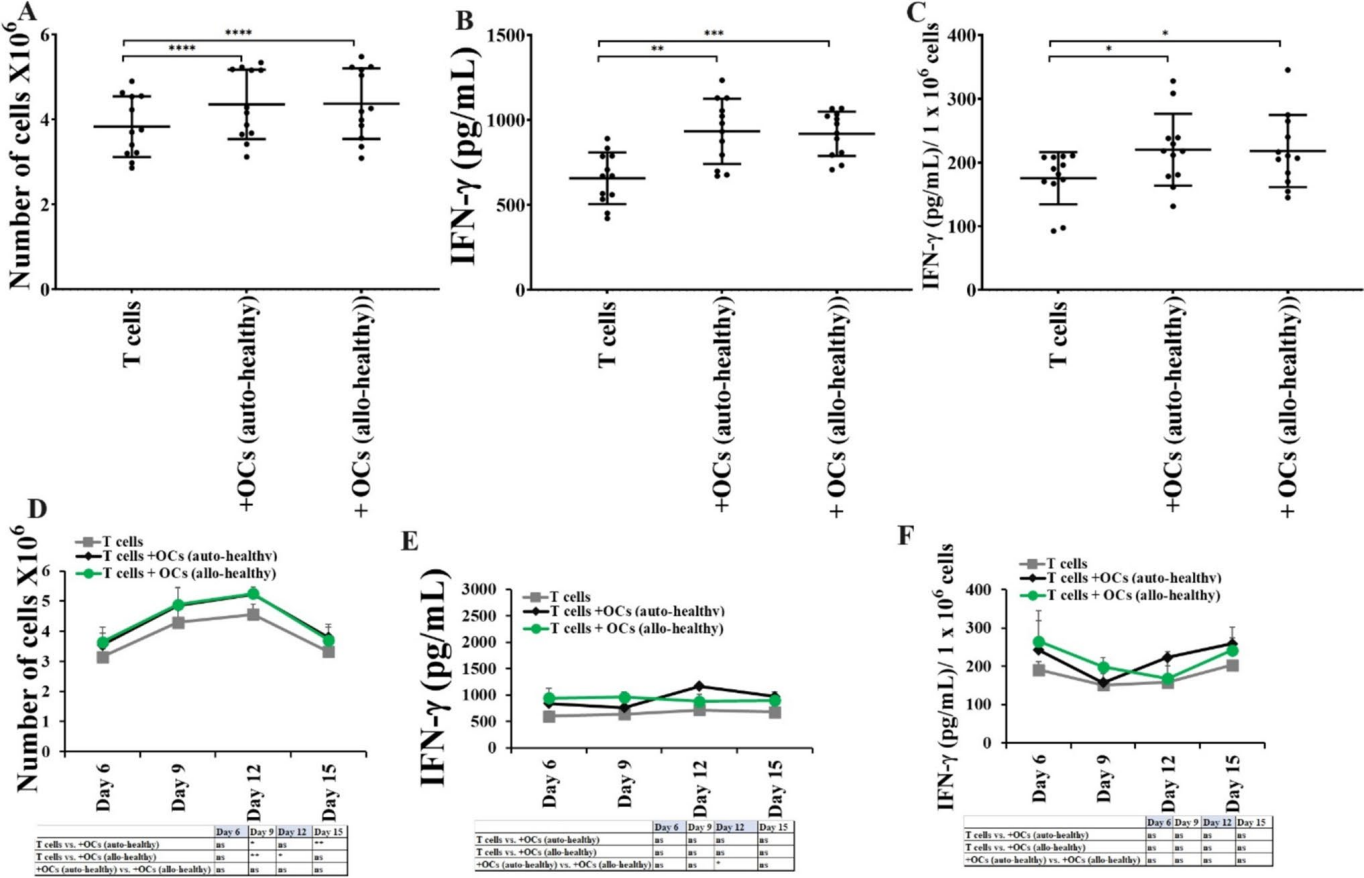
Autologous or allogeneic OCs from healthy individuals induced similar levels of expansion and functional activation in T cells. T cells (1 × 10^6^ cells/ml) from healthy individuals were treated with a combination of IL-2 (100 U/ml) and anti-CD3 (1 µg/ml)/CD28mAb (3 µg/ml) for 18 h before they were co-cultured with healthy individuals’ autologous or allogeneic OCs and sAJ2 at a ratio of 1:2:4 (OCs:T:sAJ2). On days 6, 9, 12, and 15, the T cells were counted using microscopy (n = 8) **(A, D)**. T cells were treated and cultured as described in Fig. 7A and D. The supernatants were harvested on days 6, 9, 12, and 15, and the levels of IFN-γ secretion were determined using a single ELISA (n = 8) **(B, E)**. Amounts of IFN-γ secretion shown in Fig. 7B and E were assessed based on 1 × 10.^6^ cells **(C, F)**. ****(*p* value < 0.0001), ***(*p* value < 0.001), **(*p* value 0.001–0.01), *(*p* value 0.01–0.05)

Cancer patients’ T cells represent memory type phenotype, increased surface expression of activated T cell markers, and decreased surface expression of naïve T cell markers were seen in cancer patient T cells (Fig. S8). Also, we found decreased cell expansion and IFN-γ secretion in T cells from cancer patients compared to those from healthy individuals (Fig. 5). To determine if we can reverse this inhibited T cell function, we cultured T cells from healthy individuals and cancer patients with healthy allogeneic OCs and DCs. Inhibition of cell expansion and function of T cells was not revered by either OCs or DCs (Fig. 5). We next compared autologous vs. allogeneic OCs-induced activation in healthy individual and cancer patients derived T cells, and no significant differences were seen in allogeneic or autologous OCs induced activation in T cells (Fig. 6). When we cultured healthy individual T cells with either autologous or allogeneic healthy individual-derived OCs. No significant differences were observed in the levels of cell expansion (Fig. 7A, D) and IFN-γ secretion (Fig. 7B, C, E, F) in T cells activated by either autologous or allogeneic OCs.

### OCs preferentially expand and activate CD8 + T cells

**Fig. 8:**
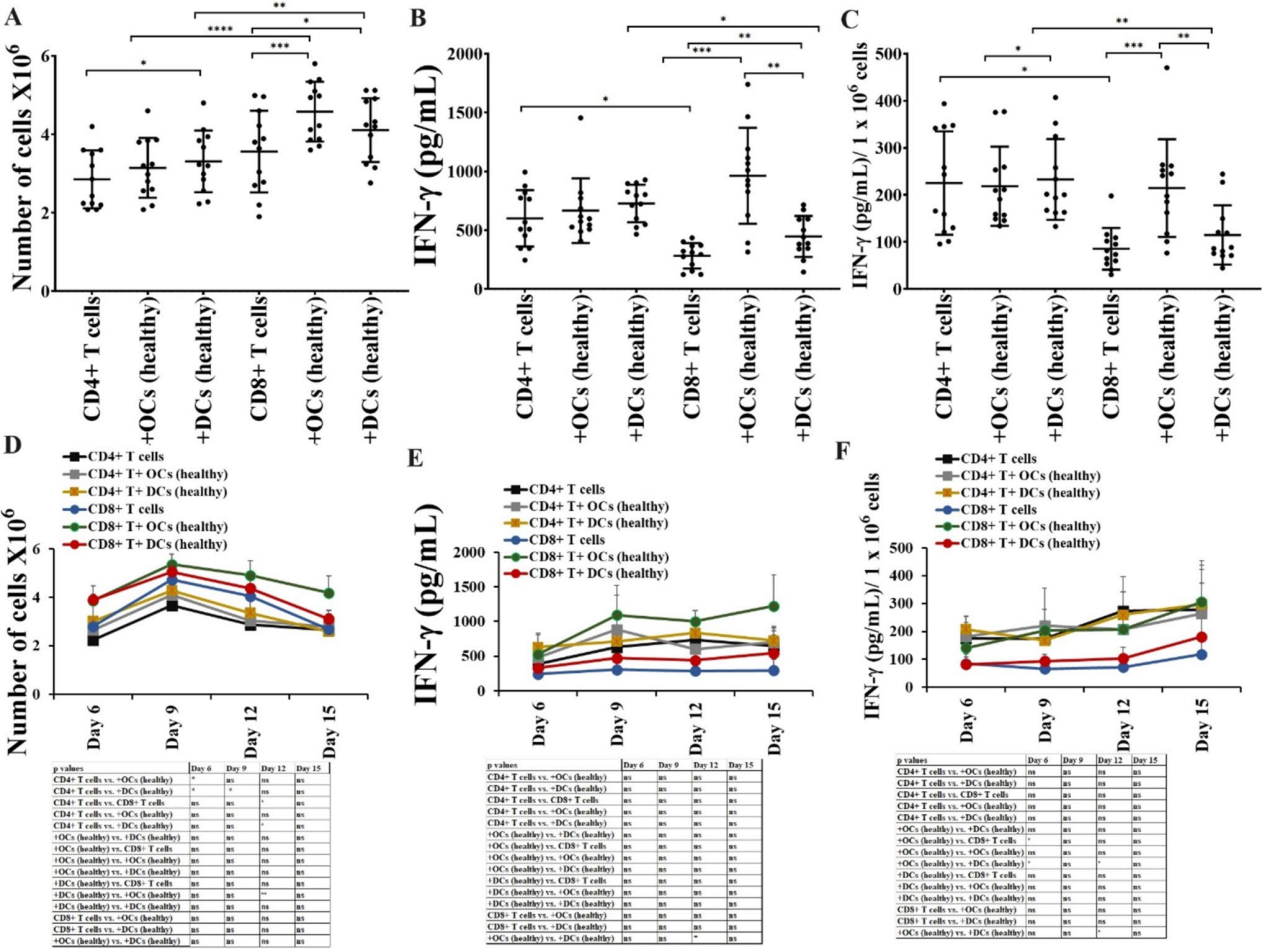
OCs preferentially expand and activate CD8 + T cells. CD4 + T cells and CD8 + T cells (1 × 10^6^ cells/ml) from healthy individuals were treated with a combination of IL-2 (100 U/ml) and anti-CD3 (1 µg/ml)/CD28mAb (3 µg/ml) for 18 h before they were co-cultured with healthy individuals’ OCs or DC and sAJ2 at a ratio of 1:2:4 (OCs or DCs:T:sAJ2). On days 6, 9, 12, and 15, the CD4 + T and CD8 + T cells were counted using microscopy (n = 12) **(A, D)**. CD4 + and CD8 + T cells were treated and cultured as described in Fig. 8A and D. The supernatants were harvested on days 6, 9, 12, and 15, and the levels of IFN-γ secretion were determined using a single ELISA (n = 12) **(B, E)**. Amounts of IFN-γ secretion shown in Fig. 8B and E were assessed based on 1 × 10.^6^ cells (n = 12) **(C, F)**. ****(*p* value < 0.0001), ***(*p* value < 0.001), **(*p* value 0.001–0.01), *(*p* value 0.01–0.05)

CD4 + T cells and CD8 + T cells from healthy individuals were cultured with healthy individuals’ OCs or DC. A slightly higher level of cell expansion was seen in CD8 + T cells alone compared to CD4 + T cells alone (Fig. 8A, D). Both OCs and DCs were found to induce higher expansion of CD8 + T cells compared to CD4 + T cells, however, OCs in comparison to DCs induced higher expansion of CD8 + T cells (Fig. 8A, D). We then compared the secretion of IFN-γ by CD4 + T and CD8 + T cells alone or in the presence of OCs or DCs. We observed higher levels of IFN-γ in CD4 + T alone compared to CD8 + T cells alone, and similar levels of increase in the secretion levels of IFN-γ were seen by DCs in both CD4 + and CD8 + T cells (Fig. 8B, C, E, F). However, OCs induced significantly increased levels of IFN-γ secretion in CD8 + T cells compared to CD4 + T cells, and also OCs-activated CD8 + T cells secreted higher IFN-γ compared to DCs-activated CD8 + T cells (Fig. 8B–C, E–F).

## Discussion

There are ample reports on DCs since these cells are responsible for activating T cells in an antigen-specific manner through peptide processing and presentation. However, we are just starting to appreciate the significance of OCs in activating immune function. In addition, we do not know whether there are significant differences between DCs and OCs on immune activation per cell basis. In this paper, we determined the function of OCs and DCs against NK and T cells in the setting of both autologous and allogeneic systems to assess which population is superior in activating NK and T cells.

When determining the function of NK cells against both OCs and DCs it is apparent that OCs have a superior ability to activate NK cells compared to DCs on per cell basis. OCs not only increase the expansion of NK cells but are also capable of increasing the function of NK cells which surpasses those mediated by the DCs. Although OCs can also increase the expansion and activation of patient-derived NK cells, the levels remain inferior to those obtained in the culture of healthy donor NK cells with healthy donor OCs. Both NK cells and OCs from patients have defects which is reflected in the decreased activation when either NK cells or OCs from patients were used as compared to those of healthy individuals [33]. A similar effect is seen by patient-derived DCs when compared to DCs isolated from healthy individuals, indicating defects in patient NK or OCs and DCs. We have previously shown that OCs from patients have lower levels of activating receptors, therefore, the defect could be at the levels of the expression of such receptors and ligands that are responsible for the activation of NK cells (Fig. S6) [33]. This receptor-ligand interaction contributes to OCs-induced activation in NK cells via cell-to-cell contact. We attempted to expand NK cells using the supernatant from OCs but we failed to achieve higher levels of NK cell expansion and functional activation indicating that cell-to-cell interaction is crucial for NK cell stimulation by OCs (manuscript in press). Next, we assessed whether there are differences in the activation of NK cells if either autologous or allogeneic OCs were used. The data indicates that no significant differences could be ascertained when either autologous or allogeneic OCs were used to activate NK cells.

When the functions of T cells were compared to NK cells, several important factors could be observed. First, both OCs and DCs were capable of activating T cells, similar to those seen by the NK cells. However, unlike NK cells no significant differences could be seen between the activating capacity of OCs compared to DCs. Patient T cells cultured with either OCs or DCs mediated lower activation when compared to T cells obtained from healthy individuals cultured with OCs or DCs. The decrease in T cell activation was less than that seen with the NK cells, therefore, it appears that T cells are less inactivated in the patients when compared to NK cells. Autologous and allogeneic OCs mediated similar levels of T cell activation similar to NK cells. When activating CD4 + T cells or CD8 + T cells with OCs, only CD8 + T cells were able to expand, and increase function when compared to CD4 + T cells, whereas DCs activated both CD4 + T and CD8 + T cells to similar extents. Therefore, it appears that cytotoxic cells such as NK cells and CD8 + T cells are preferentially activated significantly by the OCs. Whether OCs have been specialized to derive preferentially the activation of cytotoxic cells should await future investigation. Such findings are very important since one can derive the activation of helper or cytotoxic T cells and NK cells depending on whether OCs or DCs are used for the activation. Since T cells are inactivated less in cancer patients, one can design a strategy to use autologous T cells for engineering purposes and allogeneic NK cells to avoid graft vs. host disease (GVHD).

At the moment it is not clear whether OCs similar to DCs are able to present peptide antigens to specifically activate antigen specific T cells. OCs have in general lower levels of MHC class I and class II [27] and they will likely activate MHC class I and II expression at lower levels when activated as compared to DCs [44]. However, it has been shown that cross-presentation by OCs induces FOXP3 in CD8 + T cells [45]. The authors observed increases in IL-2, IL-6, and IFN-γ as well as the increase in proliferation of CD8 + T cells in OT-I transgenic mice in the presence of ovalbumin (OVA) [45]. In another study the authors demonstrated that OCs can function as antigen-presenting cells and activate both CD4 + and CD8 + T cells in an MHC-class I restricted fashion [46]. Therefore, this aspect of OCs has received relatively less attention in recent years. Whether there are quantitative and qualitative differences between antigen presentation between OCs and DCs, will require careful future investigations. However, one thing is clear OCs have a predilection to activate killer immune effectors, whereas DCs activate both CD4 and CD8 + T cells (Figs. 1, 8). These differences may have a significant physiological implication since DCs and OCs are found in different niches in tissues.

We have previously shown that NK cells target CD4 + T cells and result in the expansion of CD8 + T cells [30]. Whether CD4 + T cells have in general lower survival capability even in the presence of OCs awaits future investigations.

Since OCs are found in the bone marrow and the bone microenvironment, their ability to increase and activate CD8 + T cells could have an important physiological function in the remodeling of the bone. Indeed, it is possible that the bone-resorbing ability of OCs could be directly through OCs and indirectly through the activation of CD8 + T cells to eliminate dead and dying osteocytes in order to maintain a balance of bone formation and bone resorption.

Overall, our data demonstrates several important differences between the activating capacity of OCs and DCs on NK and T cells. One can leverage such differences to specifically activate certain subpopulations of T and NK cells to deliver effective function in cancer or other autoimmune diseases. Indeed, we have leveraged the activating capacity of OCs on NK cells to engineer supercharged NK cells which were shown to have significant tumor-killing capability by decreasing the size and the volume of tumors in vitro and in vivo in humanize-BLT mouse model systems [34, 47, 48], in addition to their use in cancer patients (manuscript submitted).

## Supplementary Information

Below is the link to the electronic supplementary material.

Supplementary file1 (DOCX 1377 KB)

## Data Availability

No datasets were generated or analysed during the current study.

## Abbreviations

DCs: Dendritic cells
IFN-γ: Interferon-gamma
MHC: Major histocompatibility complex
NK cells: Natural killer cells
OCs: Osteoclasts
OSCSCs: Oral squamous cancer stem cells
PBMCs: Peripheral blood mononuclear cells
rhIL-2: Recombinant human IL-2
