# Supplementary material for: Distinct profiles of osteoclast and dendritic cell-mediated expansion and functional activation of NK and T cells
Source: Cancer Immunol Immunother. 2025 Mar 1;74(4):127. doi: 10.1007/s00262-025-03956-x (PMC11872835; doi:10.1007/s00262-025-03956-x)
Supplement: Supplementary file 1 — Supplementary file1 (DOCX 1377 KB) [file 262_2025_3956_MOESM1_ESM.docx]

**Supplementary Data**

**Figure S1:** NK cells from healthy individuals (1x10^6^ cells/ml) were treated with a combination of IL-2 (1000 U/ml) and anti-CD16mAb (3 µg/ml) for 18 hours before they were co-cultured with autologous OCs or DCs in the presence of sAJ2 at 1:2:4 ratios (DCs or OCs:NK:sAJ2). The supernatants were harvested on day 18 of the co-cultures, and the amounts of IFN-γ secretion were determined using the multiplex assay (n=3)**.**

**Figure S2**: Monocytes were purified from human PBMCs and were cultured with GM-CSF (150 ng/ml) and interleukin (IL)-4 (50 ng/ml) for 8 days to generate DCs. To generate OCs, monocytes were cultured in alpha-MEM media containing M-CSF (25 ng/ml) and RANKL (25 ng/ml) for 21 days. OCs and DCs were used to analyze ULBPs, KIR2, KIR3, killer cell lectin-like receptor G1 (KLRG1), and MICA/B surface expressions using PE-conjugated antibodies and flow cytometric analysis. IgG2 isotype control antibody was used as control.

**Figure S3:** NK cells from healthy individuals (1x10^6^ cells/ml) were treated with a combination of IL-2 (1000 U/ml) and anti-CD16mAb (3 µg/ml) for 18 hours before they were co-cultured with allogeneic healthy and pancreatic cancer patient OCs in the presence of sAJ2 at 1:2:4 ratios (OCs:NK:sAJ2). The supernatants were harvested on day 18 of the co-cultures, and the amounts of IFN-γ secretion were determined using the multiplex assay (n=2)**.**

**Figure S4:** NK cells from healthy individuals and cancer patients were used to analyze CD16+CD94+ and CD16+NKG2D+ surface expressions using flow cytometric analysis. IgG2 isotype control antibody was used as control.

**Figure S5**: NK cells from healthy individuals and cancer patients (1x10^6^ cells/ml) were treated with a combination of IL-2 (1000 U/ml) and anti-CD16mAb (3 µg/ml) for 18 hours before they were co-cultured with allogeneic healthy OCs in the presence of sAJ2 at 1:2:4 ratios (OCs:NK:sAJ2). Surface markers were analyzed on day 21 expanded NK cells using flow cytometer.

**Figure S6**: Osteoclasts were generated using healthy individual and pancreatic cancer patient monocytes. On day 15 of osteoclast generation, osteoclasts of healthy individuals and cancer patients were analyzed for surface expression as shown in the figure using flow cytometry. IgG isotype control was used to assess non-specific binding. One of 12 representative experiments is shown in the figure.

**Figure S7**: T cells (1x10^6^ cells/ml) from healthy individuals were treated with a combination of IL-2 (100 U/ml) and anti-CD3 (1 µg/ml)/CD28mAb (3 µg/ml) for 18 hours before they were co-cultured with healthy individuals’ OCs or DC and sAJ2 at a ratio of 1:2:4 (OCs or DCs:T:sAJ2). On days 6, 9, 12, and 15, the percentages of CD4+ and CD8+ T cells in the culture were analyzed using flow cytometric analysis. IgG2 isotype control antibody was used as control.

**Figure S8**: T cells from healthy individuals and cancer patients were used to analyze memory and naïve T cell phenotype using flow cytometric analysis. IgG2 isotype control antibody was used as control.

**Figure S1**


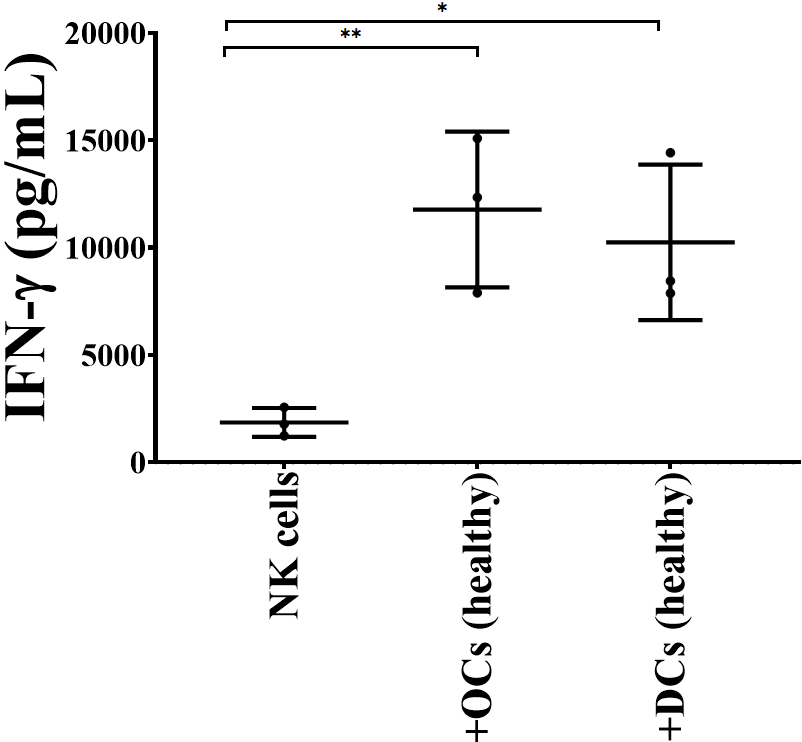


**Figure S2**


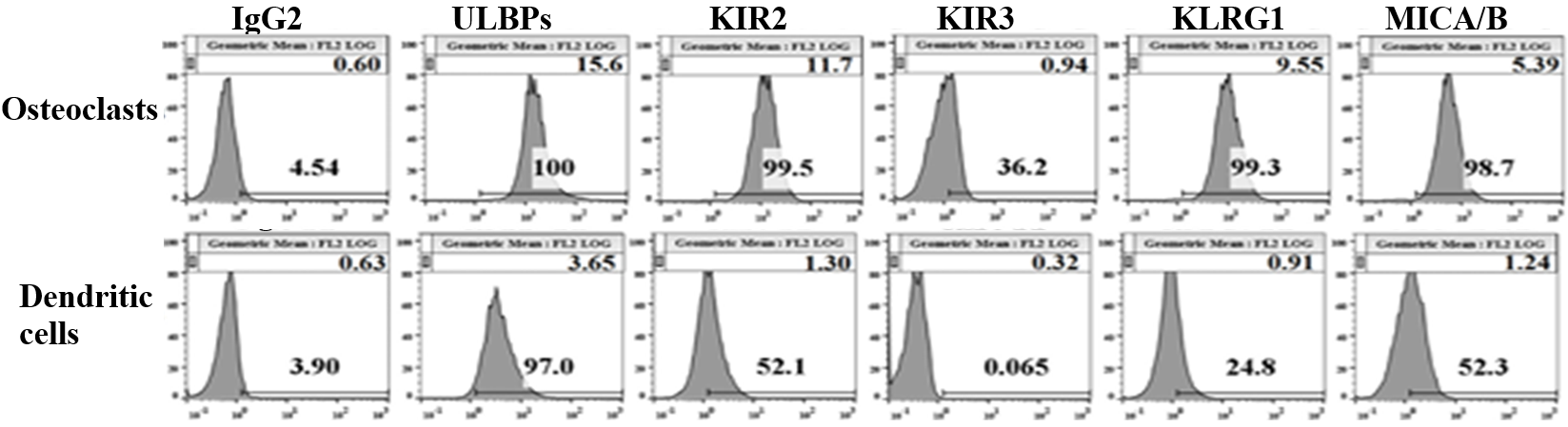


**Figure S3**


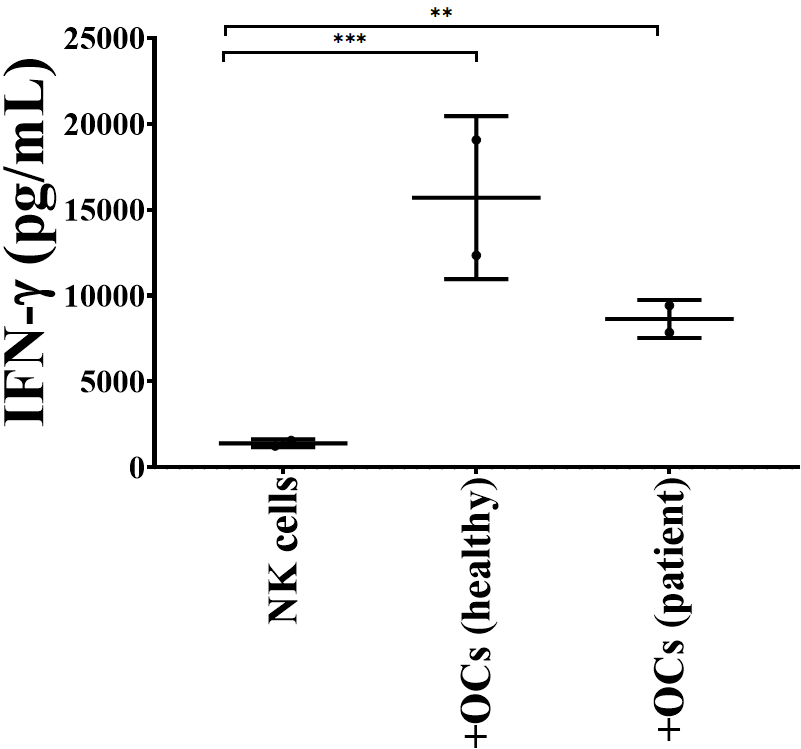


**Figure S4**


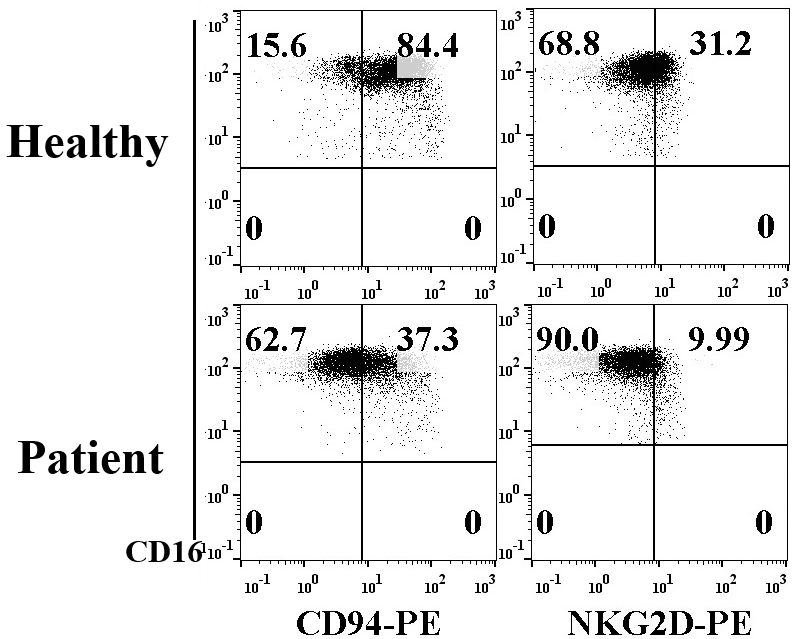


**Figure S5**

**
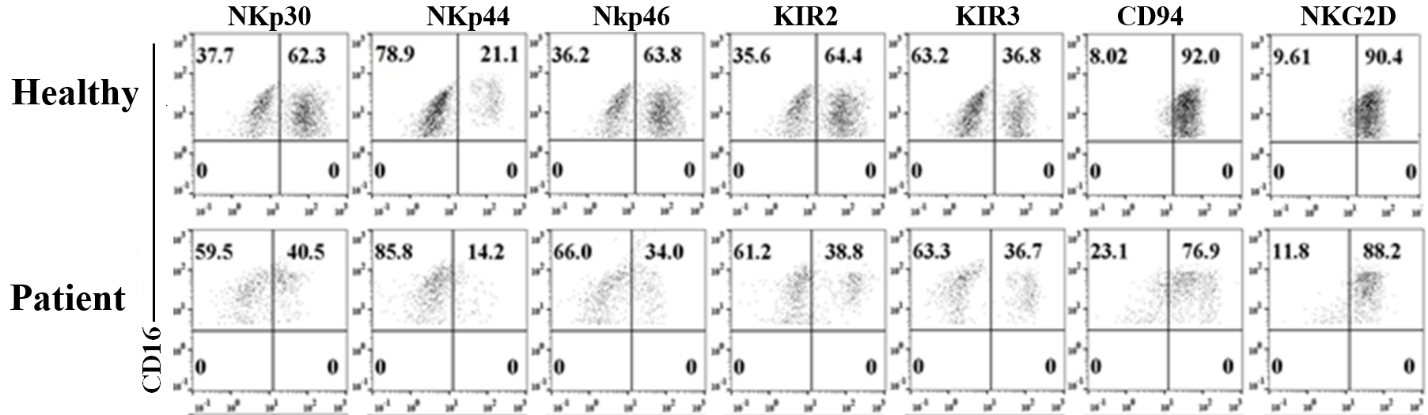
**

**Figure S6**


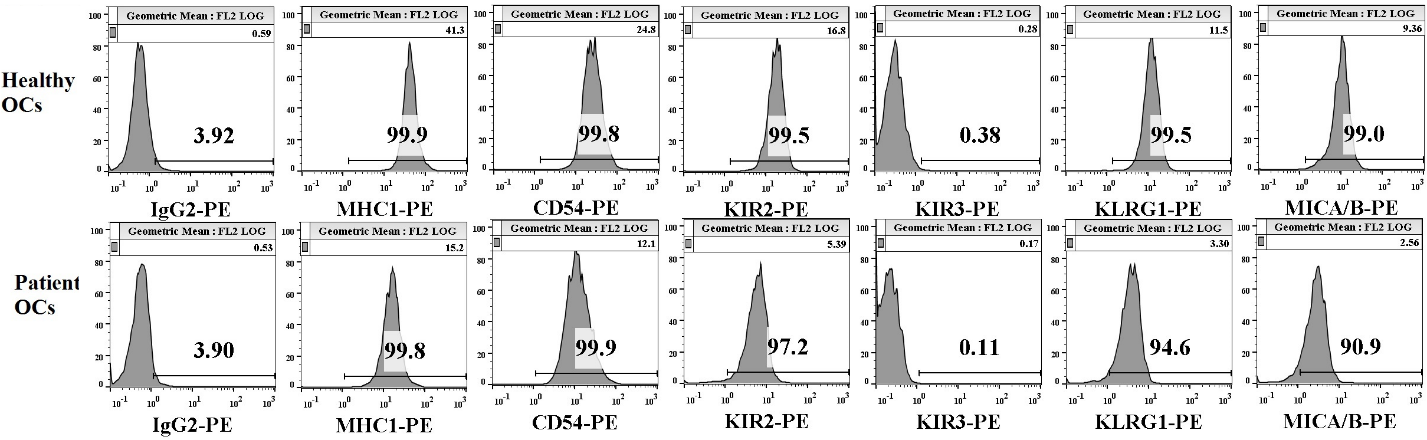


**Figure S7**

**
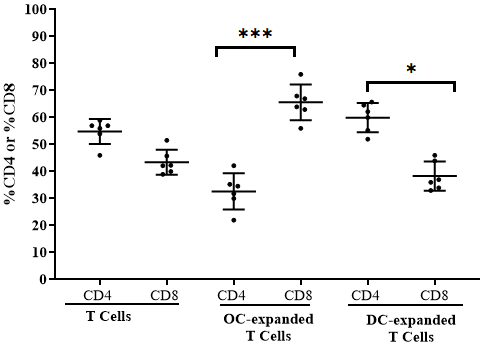
**

**Figure S8**

**
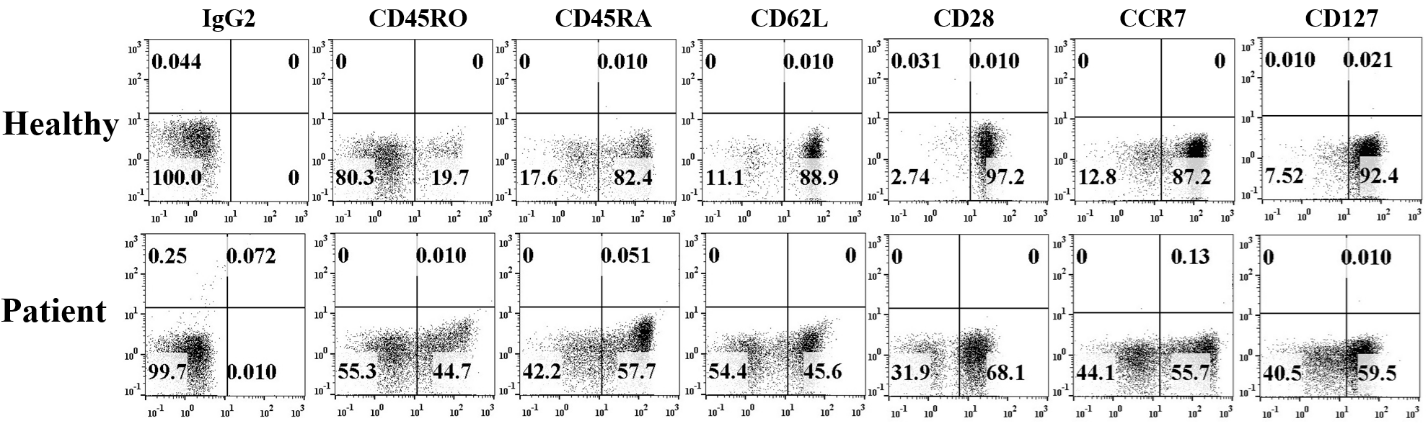
**
